# Posture, Flexibility and Grip Strength in Horse Riders

**DOI:** 10.2478/hukin-2014-0066

**Published:** 2014-10-10

**Authors:** Sarah Jane Hobbs, Joanna Baxter, Louise Broom, Laura-Ann Rossell, Jonathan Sinclair, Hilary M Clayton

**Affiliations:** 1 Centre for Applied Sport and Exercise Sciences, University of Central Lancashire, Preston, UK.; 2 Craven College, Skipton, UK.; 3 Personal Best, Yorks, UK.; 4 Myerscough College, Bilsborrow, UK.; 5 Sport Horse Science, LC, MI, USA.

**Keywords:** asymmetry, leg length, equine, back pain, laterality, equestrian

## Abstract

Since the ability to train the horse to be ambidextrous is considered highly desirable, rider asymmetry is recognized as a negative trait. Acquired postural and functional asymmetry can originate from numerous anatomical regions, so it is difficult to suggest if any is developed due to riding. The aim of this study was therefore to assess symmetry of posture, strength and flexibility in a large population of riders and to determine whether typical traits exist due to riding. 127 right handed riders from the UK and USA were categorized according to years riding (in 20 year increments) and their competition level (using affiliated test levels). Leg length, grip strength and spinal posture were measured and recorded by a physiotherapist. Standing and sitting posture and trunk flexibility were measured with 3-D motion capture technology. Right-left differences were explored in relation to years riding and rider competitive experience. Significant anatomical asymmetry was found for the difference in standing acromion process height for a competition level (−0.07±1.50 cm Intro/Prelim; 0.02±1.31 cm Novice; 0.43±1.27 cm Elementary+; p=0.048) and for sitting iliac crest height for years riding (−0.23±1.36 cm Intro/Prelim; 0.01±1.50 cm Novice; 0.86±0.41 cm Elementary+; p=0.021). For functional asymmetry, a significant interaction was found for lateral bending ROM for years riding x competition level (p=0.047). The demands on dressage riders competing at higher levels may predispose these riders to a higher risk of developing asymmetry and potentially chronic back pain rather than improving their symmetry.

## Introduction

Studies investigating rider posture during horseback riding are beginning to emerge in the literature with a common theme being asymmetry in riders (Licka et al., 2004; De Cocq et al., 2009; Symes and Ellis, 2009; Roepstorff et al., 2009; De Cocq et al., 2010). Since the ability to train the horse to be ambidextrous is considered highly desirable, rider asymmetry is recognized as a negative trait. In studying rider asymmetry, the challenge is not only to measure right-left differences, but also to determine whether they are due to structural differences in anatomical dimensions, inherent laterality, or whether they have been acquired as a consequence of riding. Acquired asymmetry in riders may develop from functional or dynamical differences when performing habitual tasks during riding, which are exacerbated through repetition and/or pain avoidance, resulting in an accumulation of postural defects.

In riding, the complexities of functional and dynamical asymmetry are potentially increased as both horse and rider can be affected, and this may also be magnified by the ability of the rider. Improved harmony between the horse and the rider has been reported in more experienced riders (Peham et al., 2001; Licka et al., 2004; Peham et al., 2004; Byström et al., 2009), so it is reasonable to expect that highly experienced riders and their horses would be more symmetrical. There is evidence, however, to suggest that even highly trained dressage riders find it difficult to form an accurate perception of rein tension in left and right hands, due to the amount of sensory information and external stimuli they are receiving simultaneously (Terada et al., 2006).

Laterality due to dominance of one side of the body is another cause of asymmetry in humans. Substantial inter-limb differences in control of limb dynamics are associated with handedness in adults (Bagesterio and Sainburg, 2002). In sport, differences in performance due to limb dominance are commonly reported in the literature (Kellis and Katis, 2007). Typically, the preferred limb is used for mobilization whereas the non-preferred limb is used for support (Sadeghi et al., 2000). Postural adaptations, such as asymmetry of the pelvis are also found (Bussey, 2010). It appears that modified tension patterns within the musculo-ligamentofascial apparatus stabilizing the pelvis, due to left-right differences in the mechanical loads transmitted through it, increase the potential of developing static pelvic asymmetry (Gnat and Saulicz, 2008). During riding, propulsive forces from the horse’s limbs are transmitted to the rider (von Peinen et al., 2009) and asymmetry in these forces due to left-right differences in strength or subclinical lameness could affect loading symmetry on the rider’s pelvis. The magnitude and prevalence of pelvic asymmetry in unilateral sports was, however, found to be greater than in bilateral sports (Bussey, 2010).

Pelvic asymmetry in the frontal plane may also occur due to leg length discrepancies, whereby paired limbs are noticeably disproportionate, although agreement of what constitutes a clinically significant length difference sufficient to induce postural changes is currently lacking (Beattie et al., 1990; Gibbons et al., 2002; Gurney, 2002). When these changes are induced temporarily using a lift in the shoe, in addition to inducing pelvic tilt, they are also reported to cause pelvic torsion and increased lateral flexion of the trunk towards the side of the limb that has been lifted (Young et al., 2000). Symes and Ellis (2009) found a relationship between asymmetry in shoulder rotation during riding and a leg length discrepancy during standing. They used the palpation meter (PALM) method (Petrone et al., 2003) to measure leg length indirectly, so it is possible that pelvic asymmetry rather than a leg length inequality influenced shoulder rotation.

Subtle anatomic abnormalities in the pelvis are also associated with altered mechanics in the lumbar spine, often due to lower back pain (Al-Eisa et al., 2006). Back pain has been linked to sagittal plane spinal posture (Norris and Berry, 1998), which was suggested to be manifested as a loss of lordosis and an anterior shift in the sagittal vertical axis of the body. These postural changes were related to the development of degenerative changes in the spine (Roussouly et al., 2005). It is unclear though whether individuals develop altered static and dynamic loading patterns prior to or subsequent to the first bout of pain (Jones et al., 2012). Subjects with lower back pain are also reported to have deficits in standing and seated balance and automatic postural coordination (Cacciatore et al., 2005). In riders the incidence of lower back pain was reported to be higher than the incidence found in the general population (Kraft et al., 2007; 2009), but it is unclear whether risks are discipline specific (Quinn and Bird, 1996; Kraft et al., 2009).

Symmetry in riders is desired, but in a small group of riders anatomical and functional asymmetry has previously been identified (Symes and Ellis, 2009). Testing a larger population of riders to establish if particular asymmetry is commonly found, if anatomical or/and functional asymmetry is more prevalent in less experienced riders compared to higher level riders (Peham et al., 2001; Licka et al., 2004; Peham et al., 2004; Byström et al., 2009) or riders who have been riding for a greater number of years (Quinn and Bird, 1996), would make an important contribution to current knowledge. The aim of this study was therefore to determine whether anatomical asymmetry (leg length, pelvis and shoulder height), functional asymmetry (trunk lateral bending and axial rotation range of motion (ROM) during sitting) and dynamical asymmetry (grip strength) were prevalent in a larger population of riders and to determine whether typical traits exist due to riding. It was hypothesised that a greater number of years riding in riders with less ability would lead to an accumulation of different types of asymmetry, together with increased prevalence of postural defects and pain. If this is indeed the case, further studies would be warranted to develop educational strategies to decrease these risks.

## Material and Methods

Ethical approval was obtained for this study from University of Central Lancashire under the approval number PSY1011104. Written informed consent was obtained from all riders prior to commencement of the study.

### Participants

The study group was comprised of 132 females and 2 males that attended a British Dressage Camp in the UK in 2011 or that attended a test event at the Michigan State University in the USA in 2011. Information was provided to participants prior to attending each testing event and participants were recruited on a voluntary basis. Of the group, 127 riders were right handed, 5 were left handed and 2 were ambidextrous. As the group was not typically representative of the normal population in relation to handedness (Annett, 1967), only data from the right handed participants was retained. This also allowed inherent right-handed traits to be considered in the analysis of results. The statistics for the group are shown in Table 1. The background of each participant was obtained with the use of a questionnaire to determine their injury history, prevalence of pain, their lifetime involvement with riding and other sports and their hand dominance. A competition level was categorized based on the level ridden in competition according to the British Dressage or the United States Dressage Federation tests: 1) intro/prelim or training/first; 2) novice or second, 3) elementary or third level and above. Riding in years was categorized as: 1) 1–19, 2) 20–39, 3) 40+. Body height was measured in standing posture against a wall and body mass was measured with a weighing scale (Salter, UK). Leg length was measured in a prone position with a neutral pelvis. Measurements were taken three times on each leg in a random order from the anterior superior iliac spine (ASIS) to the distal edge of the medial malleolus using a flexible tape measure (Anderson et al., 2005). This method has acceptable validity and reliability as a screening tool for assessing leg length discrepancy provided an average of two tape measurements is used (Gurney, 2002).

### Procedures

**Figure 1:**
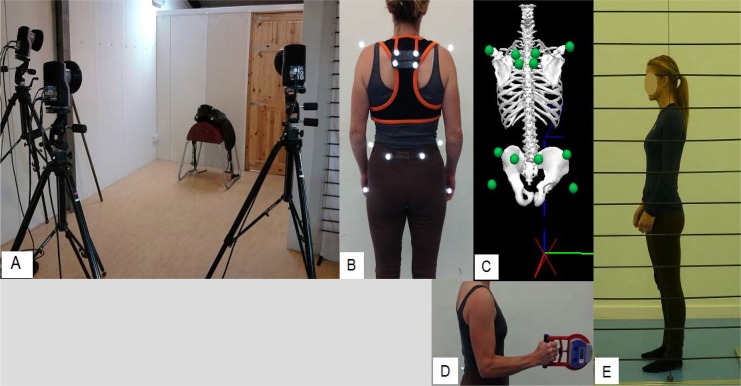
**Images from testing.** *A) Motion capture set up, B) marker set for posture and trunk flexibility tests, C) 3-D reconstruction of the trunk and pelvis, D) grip strength test, E) posture profile of a rider with kyphotic-lordotic posture.*

Images from testing are shown in Figure 1. Sagittal plane images of standing posture were captured using the method described by Norris and Berry (1998). The overall postural type was categorized (Norris and Berry, 1998; Smith et al., 2008) by a chartered physiotherapist. Grip strength was measured using a grip strength dynamometer (Takei, Japan). Participants were asked to grip the dynamometer as hard as they could whilst flexing their elbow at 90 degrees (Hanten et al., 1999). Three trials were performed in a random order between left and right arms.

The UK studies used a four camera infrared motion capture system for which the error in a linear measurement of 750.5 mm was <2.3mm (Qualisys Capture Systems, Gothenburg, Sweden) and the US studies used a ten camera infra-red motion capture system for which the error in a linear measurement of 1000 mm was <0.8 mm (Motion Analysis Corp., Santa Rosa, CA). Participants were recorded during seated posture and whilst performing trunk motion exercises. The systems were calibrated in order that a horse model with a saddle could be placed along one of the horizontal axes, allowing absolute positions relative to the laboratory coordinates to be measured. Of the participant group, 94 riders (1 male, 93 female), age 38.7 ± 10.8 years participated in standing and sitting postural measurements and trunk ROM tests. Retro-reflective markers were attached to the left and right acromion process, iliac crest, posterior superior iliac spine (PSIS), greater trochanter and a cluster of four markers was firmly attached in the upper thoracic region. A standing trial was captured initially with participants in the anatomical position in order for the tracking markers to be referenced to the anatomical markers. All markers remained in place for the duration of the testing.

Markers attached to the participants were then captured during sitting in their normal riding posture on a dressage saddle that was secured to a horse model. To measure trunk flexibility a lightweight wooden pole was placed across the shoulders to prevent excessive motion of the shoulder girdle. Participants remained seated on the saddle horse and performed slow left and right lateral bending and left and right rotation movements to the end of their ROM returning each time to a neutral position. Three trials for each movement were captured at 100 Hz in a random order using the same slow movement pattern for each movement (as instructed by the investigator), based on the procedures used by Al-Eisa et al. (2006). Markers tracking the trunk were identified in the software Qualisys Track Manager (Qualisys Capture Systems, Gothenburg, Sweden) and Cortex (Motion Analysis Corp., Santa Rosa, CA) then exported into Visual 3D (C-Motion Inc., Germantown, MD).

A kinematic model was created for the trunk and pelvis and applied to the sitting posture and trunk flexibility data. The acromion process and iliac crest markers were used to define the proximal and distal ends of the trunk and the cluster of four markers on the upper thorax were used to track trunk movements. The iliac crest and greater trochanter markers defined the proximal and distal ends of the pelvis and these markers together with the PSIS markers were used to track the pelvis. A 4^th^ order Butterworth filter (Robertson and Dowling, 2003) with cut off frequency of 5 Hz was applied to markers tracking trunk motion during the trunk flexibility tests to remove higher frequency noise within the data. A 5 Hz cut off was chosen as this retained 95% of the signal power. For standing and seated posture absolute position of the markers on the left and right acromion processes and iliac crests in the vertical direction were extracted. For trunk lateral bending and rotation, the range of motion of the trunk relative to the pelvis was extracted using an XYZ Cardan sequence, where X was flexion-extension, Y was lateral bending and Z was rotation. The sign convention was based on the right handed rule: in lateral bending right shoulder downwards was positive (right lateral bending) and in rotation right shoulder rotating anticlockwise when viewed from above was positive (right rotation).

### Statistical Analysis

Two factors were investigated: number of years riding (three levels) and competition experience (three levels). For the measured variables (leg length, grip strength, height of the acromion processes and iliac crests during standing and seated posture, lateral bending ROM and rotation ROM) the magnitudes for right (+) and left (-) sides were determined and the absolute difference between right and left sides was calculated (right – left). Data was tested for normality using Kolmogorov-Smirnov normality tests. All variables were normally distributed, except for leg length, which was transformed. To study the prevalence of acquired asymmetry in riders a 2 × 3 ANOVA was used to determine significant differences between two factors: years riding (× 3 levels) and competition experience (× 3 levels) with age included in all tests as a covariate except for leg length. Leg length was considered to be a skeletal difference and therefore was not expected to have been acquired due to age. Posture classification was tabulated in accordance with pain reported in specific parts of the body. In addition, the prevalence of pain in relation to posture (% per group) and the prevalence of postural types that were not normal (% per group) were calculated.

## Results

Statistics for the whole group are shown in Table 1. For this rider group 51% rode pure dressage only, 49% rode in dressage and other equestrian sports, 71% were currently participating or had previously participated in other non-equestrian sports or types of exercise, 55% reported having had at least one serious injury prior to commencing the study.

Significant anatomical asymmetry was found for the difference in standing acromion process height for the competition level (−0.07±1.50 cm Intro/Prelim; 0.02±1.31 cm Novice; 0.43±1.27 cm Elementary+; p=0.048) and for sitting iliac crest height for years riding (−0.23±1.36 cm Intro/Prelim; 0.01±1.50 cm Novice; 0.86±0.41 cm Elementary+; p=0.021). For functional asymmetry, a significant interaction was found for lateral bending ROM for years riding × competition level (−1.17±6.78 deg Intro/Prelim; 2.64±6.27 deg Novice; 2.37±5.05 deg Elementary+; 0.84±5.32 deg 0 to 19 yrs; 0.23±6.99 deg 20 to 39 yrs; 3.13±5.66 deg 40+years; p=0.047). No other significant differences were found between right and left sides, although grip strength was notably higher on the right for all groups. For all variables the coefficient of variability was high, see Tables 2, 3 and 4. The posture classifications of the riders according to regions of pain they reported are shown in Table 5. The prevalence of pain for riders with postural defects, which constitutes all other postures except for normal posture, and also for riders with normal posture, shown by group is reported in Table 6. A trend is seen in riders with postural defects developing back and/or neck pain with an increasing level of competition.

## Discussion

This study assessed a large sample of right-handed riders in relation to anatomical, functional and dynamical asymmetry to determine whether typical traits existed due to riding. The interaction in functional asymmetry with lateral bending ROM to the right greater for years riding and the competition level only in part supports the hypothesis, as symmetry was expected to improve with the competition level. For significant anatomical asymmetry, the mean difference in standing acromion process height increased with the competition level, which did not support the hypothesis. The mean difference in sitting iliac crest height altered from higher on the left to higher on the right with years riding, so there was evidence that sitting pelvic asymmetry may develop in riders, but this was not influenced by ability. A trend of increased prevalence of pain in riders at higher competition levels was found in riders that had postural defects, but this did not consistently increase with years riding.

The interaction in functional asymmetry for lateral bending ROM and an increase in prevalence of pain from 38% in low level riders to 59% in high level riders with postural defects may be clinically important. Symmetry in lateral bending and rotation was suggested to be clinically important within the individual, particularly for the diagnosis of lower back pain (Al-Eisa et al., 2006). Higher lumbar motion asymmetry has previously been reported in lower back pain patients compared to control subjects, although asymmetry is still evident in the normal population (Gomez, 1994; Al-Eisa et al., 2006). Pain has been reported in higher level riders (Kraft et al., 2009), which has been attributed to the requirement for them to absorb the considerable vertical movement of the centre of mass of the horse whilst sitting in an upright dressage posture (Auty, 2007). However, Kraft et al. (2009) found no conclusive evidence of pathologies in riders with lower back pain. In the context of pain, lateral bending asymmetry may be a restriction or stiffness in either vertebral or paravertebral structures (Al-Eisa et al., 2006), so it is possible that pain in riders is largely related to sub-clinical asymmetry. Higher level riders with postural defects may therefore have more difficulty absorbing the movements of the horse, resulting in greater pain and increased muscle stiffness. Conversely, pain avoidance during riding may increase the prevalence of postural defects and muscle imbalances in higher level riders. Further work is needed to investigate the cause and effect relationship between back pain and horse riding.

A greater mean standing acromion process height on the right for the most experienced rider group may also be linked to differences in lateral bending ROM. Sahrmann (2002) suggested that greater muscle development and therefore muscle stiffness on the right side would limit lateral bending to the left. Certainly, right grip strength was greater for all groups, which would be expected for a right handed population (Yielder et al., 2009) and grip strength was correlated with muscle mass (Kallman et al., 1990). Nicolay and Walker (2005) speculated that the use of the dominant hand in daily activities may train muscle fibres towards the properties of fast-twitch fibres and more efficient control of intersegmental dynamics may also alter muscle development (Bagesteiro and Sainburg, 2002). Muscle hypertrophy on the right could therefore explain the increase in acromion process height, which may also explain the reduction in left lateral bending ROM. This finding is not typical, as Kendall et al. (1983) reported that the dominant shoulder was normally positioned lower than the non-dominant shoulder in most people. The dynamic control needed to provide suitable signals to the horse whilst maintaining upright upper body posture is extremely important when riding dressage and it is known that rein tension varies between right and left hands (Kuhnke et al., 2010). Different left-right muscle recruitment patterns may be used to produce similar signals to the horse, which result in asymmetrical muscle development, asymmetrical shoulder height and asymmetry in lateral bending ROM.

Another explanation for reduced lateral bending ROM to the left would be a restriction due to a left axial rotation postural position of the trunk whilst completing the exercise (Sahrmann, 2002). Greater left axial rotation ROM was evident for 40 + years riding and the Elementary + level, although these were not significant and no interaction was found. Also, a difference in axial rotation ROM may not relate to a postural rotational position during a lateral bending exercise. Symes and Ellis (2009) found a preferred posture of right rotation of the shoulders during riding not left rotation, so again, this does not support the idea that riders may commonly have a left axial rotation postural defect. More quantitative measurements of anatomical, functional and dynamical asymmetry compared to ridden postural asymmetries are needed to understand the effects of riding on strength, posture and flexibility.

Finally, Al-Eisa et al. (2006) suggested that lateral bending ROM was highly associated with pelvic asymmetry in the normal population. As iliac crest height during sitting was only influenced by years riding and not ability it is unlikely that pelvic asymmetry influenced lateral bending ROM. Bussey (2010) suggested that pelvic asymmetry due to lateral dominance may decrease when athletes undertake bilaterally dominant activities. Evidence from this study suggests that sitting pelvis height does alter over time, but this does not necessarily constitute an improvement in symmetry. Riders are often reported to collapse their hip to one side and may show increased pressure under the saddle on the same side or on the opposite side (Clayton, unpublished). It may be surmised that riding posture and the application of signals to the horse influence static sitting posture. Gnat and Saulicz (2008) found changes in functional asymmetry of the lumbo-pelvo-hip complex following mechanical stimulation. This was attributed to a change of tension patterns within the musculo-ligamento-fascial apparatus that maintains stability of the pelvis. It is possible that together with the application of signals to the horse, the force transmission through the pelvis during riding alters the tension in the musculo-ligamento-fascial apparatus over time and, therefore, influences pelvic alignment.

Although an anatomical difference and not an acquired difference, a leg length discrepancy was not found in this rider sample, which is contrary to the findings of Symes and Ellis (2009). They reported a shorter right leg in a considerably smaller sample of riders. Discrepancies can occur through an inaccurate measurement of the limbs, depending on the method used (Sabharwal and Kumar, 2008). Our leg length findings are also supported by standing iliac crest height results, as iliac crest height was reported to provide clinically useful evidence for suspecting leg length inequality (Young et al., 2000).

The absolute difference between left and right sides for each measurement and each participant was calculated, grouped, and then used to establish asymmetry patterns in riders. Some participants tended to produce larger values to the right and other participants tended to produce larger values to the left, so the mean for each group tended towards zero and the standard deviation tended to be large. This is highlighted by the large values reported for the coefficient of variability in Tables 2, 3 and 4. Due to the large variability between riders further work should incorporate longitudinal within-rider monitoring, which may indicate better the cause and effect relationships between riding and changes in symmetry.

This study reported anatomical, functional and dynamical asymmetry in a large sample of riders. Data were collected at a number of locations in the UK and USA and as such care was taken to replicate the same procedures at each data collection session. Despite this there are differences in the accuracy of the measurements taken using motion capture techniques, as identified in the method. To reduce bias between samples and compare symmetry the difference between right and left measures for each variable were analysed. For the motion capture data markers were missing on some occasions during testing, mainly due to occlusion, so for some participants the end of range of motion could not be determined. In addition, on a small number of occasions participant information or recorded measurements were missing. Consequently, the number of samples included for each variable was not consistent throughout the study. These are reported in Tables 2, 3 and 4.

## Conclusions

Symmetry of posture, strength and flexibility was assessed in a large population of riders to determine whether typical traits existed due to riding. Lateral bending ROM to the left was reduced in higher level riders that had ridden for a longer amount of time. This may be attributed to asymmetric shoulder height, suggesting that strength and therefore muscle development is greater on the right side of the body. Alternatively there is evidence to suggest that this may relate to pain. A difference in sitting pelvic asymmetry was found for years riding, which may also have restricted lateral bending ROM in the higher level riders. The demands on dressage riders competing at higher levels may predispose these riders to a higher risk of developing asymmetry and potentially chronic back pain rather than improving their symmetry.

## Figures and Tables

**Table 1:** Descriptive statistics of the riders

	Mean
Age (years)	39.2 (12.1)
Number of years riding	26.3 (12.3)
Body height (cm)	166.0 (12.8)
Body mass (kg)	67.1 (12.8)
BMI	24.4 (4.1)
Reach distance (cm)	16.5 (8.8)

**Table 2:** Statistics for anatomical asymmetry: Mean for the left and right sides, difference (Right-Left), coefficient of variability (%COV), significance (P) and number of participants included in the group (n) and including left and right measurements for leg length, acromion process (AP) height (standing and sitting) and iliac crest height (standing and sitting). ^*^Age not used as a covariate for leg length

Leg length^*^		L (cm)	Difference (R-L) cm	R (cm)	% COV	p	n
Yrs Riding	0–19	83.4 (6.7)	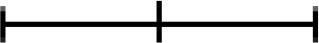	83.4 (6.8)	2050	0.676	27
	20–39	83.7 (5.8)	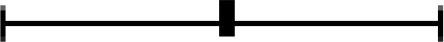	83.7 (5.9)	1450		54
	40+	84.7 (5.1)	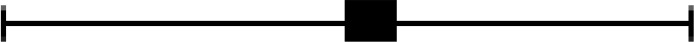	84.8 (5.2)	643		38

Comp Level	Int/pre	82.1 (6.1)	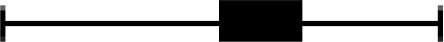	81.8 (6.1)	264	0.349	34
	Nov	83.6 (5.3)	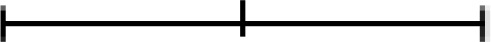	83.7 (5.4)	6300		44
	El+	85.8 (5.5)	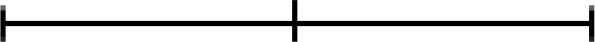	85.5 (5.6)	7700		45

**Standing AP**			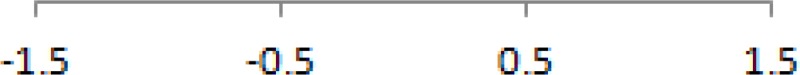		Interaction	0.924	

Yrs Riding	0–19	144 (12.7)	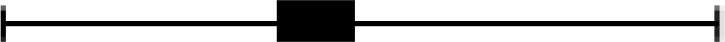	145 (12.7)	444	0.480	20
	20–39	146 (15.1)	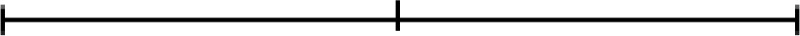	146 (15.1)	800		39
	40+	151 (13.9)	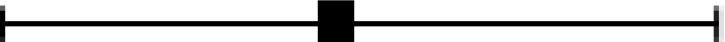	151 (13.8)	923		36

Comp Level	Int/pre	146 (12.4)	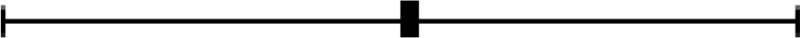	145 (12.4)	2143	**0.048**	30
	Nov	147 (14.5)	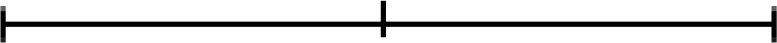	147 (14.7)	650		35
	El+	153 (18.6)	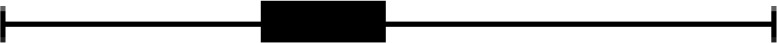	153 (18.3)	302		33

**Sitting AP**			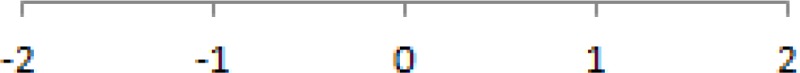		Interaction	0.792	

Yrs Riding	0–19	146 (8.6)	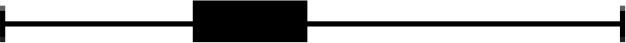	146 (8.5)	263	0.712	19
	20–39	146 (10.9)	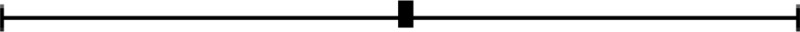	146 (11.0)	2600		40
	40+	148 (11.4)	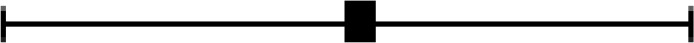	148 (11.3)	1150		36

Comp Level	Int/pre	146 (8.8)	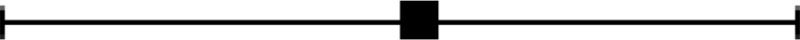	145 (8.6)	1029	0.513	30
	Nov	147 (10.8)	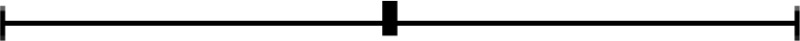	147 (10.6)	2317		35
	El+	151 (17.6)	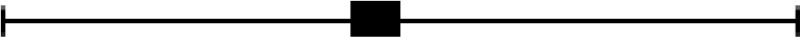	151 (18.0)	811		33

**Standing iliac**			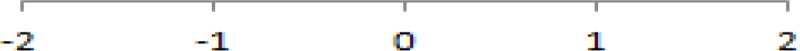		Interaction	0.891	

Yrs Riding	0–19	108 (10.6)	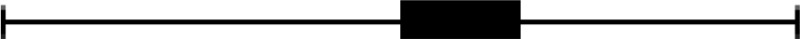	107 (10.8)	327	0.070	20
	20–39	109 (13.0)	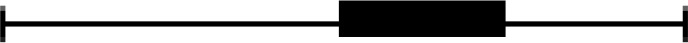	108 (13.1)	209		37
	40+	113 (12.2)	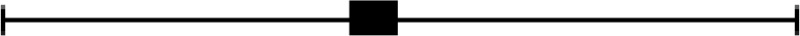	113 (12.2)	790		35

Comp Level	Int/pre	109 (10.2)	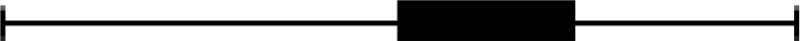	108 (10.5)	225	0.489	30
	Nov	110 (12.9)	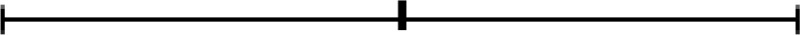	110 (13.2)	5533		33
	El+	111 (14.1)	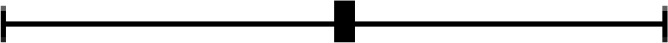	115 (17.6)	1586		32

**Sitting iliac**			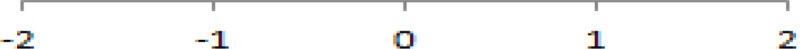		Interaction	0.212	

Yrs Riding	0–19	111 (6.2)	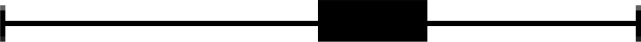	111 (6.0)	296	**0.021**	15
	20–39	112 (9.9)	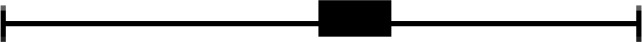	112 (9.7)	457		32
	40+	112 (10.7)	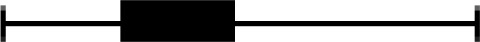	112 (9.5)	200		30

Comp Level	Int/pre	112 (6.5)	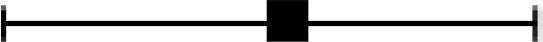	111 (6.6)	705	0.919	24
	Nov	111 (9.1)	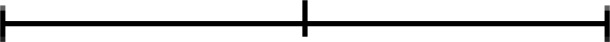	111 (9.0)	15300		28
	El+	117 (19.2)	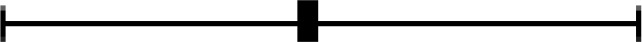	117 (19.0)	1455		28

			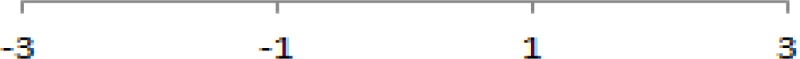		Interaction	0.925	

**Table 3:** Statistics for dynamical asymmetry: Difference (Right-Left), coefficient of variability (%COV), significance (P) and number of participants included in the group (n) and including left and right measurements for grip strength

**Grip strength**		L (kg)	Difference (R-L) kg	R (kg)	% COV	p	n
Yrs Riding	0–19	23.6 (7.9)	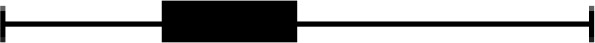	25.2 (7.8)	216	0.427	27
	20–39	24.6 (8.7)	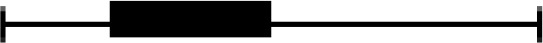	26.5 (9.1)	164		54
	40+	23.2 (10.0)	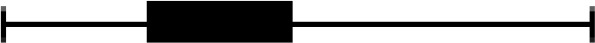	24.9 (10.7)	195		38

Comp Level	Int/pre	25.1 (8.6)	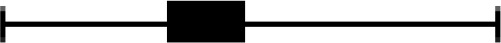	26.0 (8.9)	302	0.064	34
	nov	23.5 (7.4)	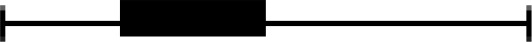	25.2 (7.5)	180		44
	el+	23.1 (10.2)	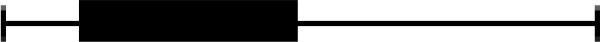	25.7 (11.0)	135		45

			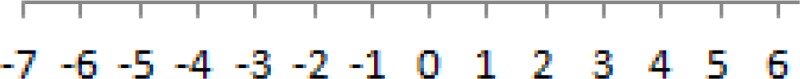	Interaction		0.653	

**Table 4:** Statistics for functional asymmetry: Difference (Right-Left), coefficient of variability (%COV), significance (P) and number of participants included in the group (n) and including left and right measurements for lateral bending and axial rotation range of motion

**Lateral bending**		L (deg)	Difference (R-L) deg	R (deg)	% COV	p	n
Yrs Riding	0–19	38.9 (8.3)	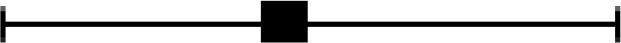	39.8 (7.3)	631	0.181	21
	20–39	37.2 (8.4)	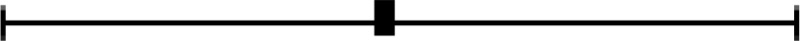	37.6 (8.9)	1795		38
	40+	34.2 (7.7)	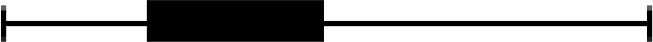	37.6 (7.0)	182		34

Comp Level	Int/pre	38.5 (8.4)	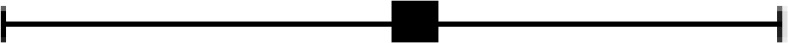	37.6 (7.8)	817	0.143	29
	nov	36.5 (8.6)	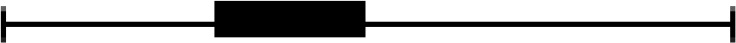	39.1 (7.2)	238		35
	el+	35.0 (7.6)	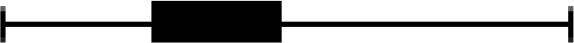	37.3 (8.5)	215		32

**Rotation**			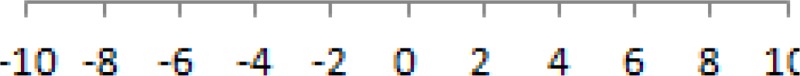	Interaction		**0.047**	

Yrs Riding	0–19	45.2 (8.6)	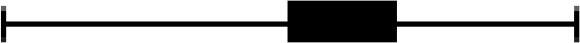	43.0 (7.5)	260	0.528	17
	20–39	40.9 (8.7)	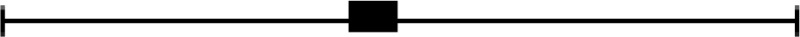	42.0 (9.1)	842		34
	40+	43.9 (7.0)	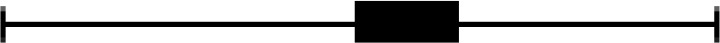	41.7 (7.5)	347		34

Comp Level	Int/pre	43.6 (7.1)	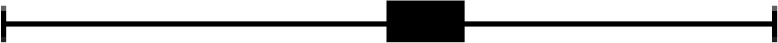	41.9 (8.3)	493	0.963	24
	nov	43.1 (9.0)	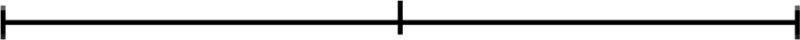	43.1 (7.5)	87000		33
	el+	43.5 (8.9)	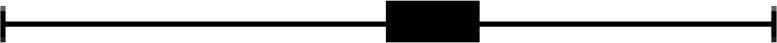	41.6 (8.1)	415		31

			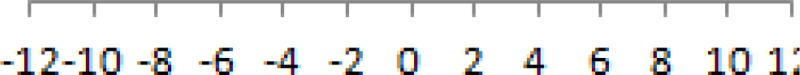	Interaction		0.813	

**Table 5:** Riders categorised by pain and posture type (n=122). Postural data was omitted from the remaining 5 riders as no information relating to pain was provided

**Posture**	**No Pain**	**Lumbar**	**Thoracic**	**Shoulder**	**Neck**	**Various**	**% of riders**
Lordotic	10	3	0	1	1	6	17
Kyphotic/lordotic	12	8	2	5	3	8	31
Kyphotic	2	2	1	0	1	2	7
Swayback	3	5	0	2	1	4	12
Normal	9	9	0	5	2	9	28
Flatback	3	1	0	1	0	1	5

**Total**	**39**	**28**	**3**	**14**	**8**	**30**	**100**

**Table 6:** Prevalence of pain for riders with postural defects (all other postures except for normal posture) and for riders with normal posture presented as a % of the group and prevalence of postural defects (all other postures except for normal posture) presented as a % of the group (n=122)

		**Level**			
**Prevalence of pain**	**Yrs Riding**	**Intro/Pre**	**Novice**	**Elem +**	**Total**
**Postural Defects**	**0–19**	23	55	33	**29**
	**20–39**	60	38	74	**56**
	**40+**	17	54	47	**45**
	**Total**	**38**	**47**	**59**	

**Normal Posture**	**0–19**	15	9	33	**11**
	**20–39**	7	24	11	**15**
	**40+**	50	31	26	**32**
	**Total**	**18**	**22**	**20**	

**Prevalence of postural defects**					
	**0–19**	54	91	67	**70**
	**20–39**	87	71	84	**80**
	**40+**	50	69	68	**63**
	**Total**	**68**	**76**	**73**	
